# Cross‐cultural, population‐based study on adolescent body image and eating distress in Japan and Finland

**DOI:** 10.1111/sjop.12485

**Published:** 2018-11-05

**Authors:** Junko Maezono, Shoko Hamada, Lauri Sillanmäki, Hitoshi Kaneko, Masayoshi Ogura, Lotta Lempinen, Andre Sourander

**Affiliations:** ^1^ Department of Child Psychiatry University of Turku Turku Finland; ^2^ The Centre for East Asian Studies (CEAS) University of Turku Turku Finland; ^3^ Department of Psychosociology School of Arts and Letters Meiji University 1‐1 Tokyo Japan; ^4^ Psychological Support and Research Center for Human Development Nagoya University Nagoya Japan; ^5^ Naruto University of Education Naruto Japan; ^6^ Department of Child Psychiatry University of Turku and Turku University Hospital Turku Finland

**Keywords:** Adolescence, body image, cross‐cultural, eating behaviour

## Abstract

This cross‐sectional population‐based survey compares the prevalence of self‐reported body image and eating distress symptoms among adolescents in Japan and Finland, and associations between emotional/behavioral problems, body image and eating distress from a cross‐cultural perspective. The study included 1,840 Japanese and 1,135 Finnish 8th grade students. The self‐reported questionnaire included the Body Image and Eating Distress Scale and Strengths and Difficulties Questionnaire (SDQ). The female adolescents from both Finland and Japan reported much greater dissatisfaction with, and concern about, their bodies than the males and Japanese females expressed even higher distress than Finnish females. High levels of body image and eating distress were associated with psychiatric problems measured with the SDQ. There was a significant three‐way interaction effect of body image and eating distress, gender and country with SDQ peer problems and prosocial behavior.

## Introduction

Body image plays an important role in adolescents’ health and development and has been defined as “a person's perceptions, thoughts, and feelings about his or her body” (Grogan, 2008). Modern culture, media and peer influences put pressure on adolescents’ body image and eating behavior and that has an impact on their health and development. Modern Western society promotes slenderness for both males and females and the ideal is that females are slim and shapely and males are slender and muscular (Grogan, 2008; O'Dea, 1995; Smolak, 2004). History shows that the factors that determine what is attractive have depended on the era, place, culture and society and the ideal that being slender is most attractive is a relatively recent phenomenon that appeared in the twentieth century (Gordon, 1990; Miller & Pumariega, 2001; Orbach, 1993). Being surrounded by the ideal that a slender body shape is preferable means that body dissatisfaction is noticeable from eight years of age and possibly even earlier (Grogan & Wainwright, 1996). Body dissatisfaction tends to be most apparent in girls when they try to lose weight, whereas in boys it can manifest itself as weight gain or weight loss (Furnham & Calnan, 1998). Previous studies have showed that body dissatisfaction is a risk factor for eating distress, depressive symptoms and low self‐esteem in both adolescent males and females (Ferreiro, Seoane & Senra, 2012; Mäkinen, Puukko‐Viertomies, Lindberg*,* Siimes & Aalberg, 2012; Paxton, Neumark‐Sztainer, Hannan & Eisenberg, 2006).

Most of the population‐based studies on body image and eating behavior have come from Western countries (Ricciardelli, 2012; Wertheim & Paxton, 2012) and the majority have focused on adults (McCabe, Fuller‐Tyszkiewicz, Ricciardelli, Skouteris & Mussap, 2012). There have been relatively few population‐based studies about eating distress among Japanese adolescents (Nakamura, Hoshino, Watanabe *et al*., 1999; Nishizawa, Kida, Nishizawa*,* Hashiba, Saito & Mita, 2003; Shirasawa, Ochiai, Hanri *et al*., 2015). Nizhizawa *et* *al*. (2003) found in a population‐based school survey that the rate of eating problems in the late 1990s among Japanese high school students was 11.2% for females and 2.4% for males. However, in the similar Japanese study from the mid‐1990s including only female high school students and using similar methodology the prevalence of eating problems was only 5.5% (Nakamura *et al*., 1999). Kayano, Yoshiuchi, Al‐Adawi *et al*. (2008) compared eating distress among 411 Japanese adolescents, with samples from 130 Indian, 135 Omani, 113 Euro‐American and 196 Filipino adolescents. It is clear that large representative population‐based sample studies comparing adolescent eating behavior problems in Japan and Western countries are needed. In general, only a few of the cross‐cultural studies have investigated body image and eating behavior by using the same methodology (Soh, Touyz & Surgenor, 2006). Cross‐cultural studies have showed that in many non‐Western countries the adoption of a Western lifestyle, and the social shift from collectivistic to individualistic values, may increase body image problems (Holmqvist & Frisen, 2010; Rieger, Touyz, Swain & Beaumont, 2001; Soh *et al*., 2006; Swami, Frederick, Aavik *et al*., 2010). A multi‐ethnic study carried out in the UK suggested that high levels of dieting for weight control and body image problems were common among adolescents in all ethnic groups (Viner, Haines, Taylor*,* Head, Booy & Stansfield, 2006).

Eating distress and body image problems among adolescents are related to race, gender, age and cultural background. Most epidemiological studies among adolescents have been conducted in Western countries. Cross‐cultural comparisons between Western and non‐Western countries are needed to bridge this gap. The current study is the first large population‐based cross‐cultural study to focus on male and female adolescents’ body image and eating distress in early adolescence using similar measurements from Japan and a Western country. The primary aim was to compare the prevalence of self‐reported body image and eating distress symptoms among adolescents in Japan and Finland. Based on the results of previous studies, we expected females to demonstrate more distress than males. Because of the large gender gap between these two countries (World Economic Forum, 2015), we hypothesized that gender differences in body image and eating distress symptoms would be more prominent among Japanese adolescents. The second aim was to study associations between psychopathology, body image and eating distress from a cross‐cultural perspective.

## Method

### Study samples and procedure

The study comprised two population‐based surveys based on 8th grade students: 1,840 (51% female) in Japan, and 1,135 (52% female) in Finland. The study samples in both countries were collected from non‐metropolitan areas. Students who were physically or mentally disabled were excluded. The questionnaires were as similar as possible to each other, subject to some cultural considerations. They were approved by the school authorities and were distributed to students by their teachers in the classroom.

The ethical standards from 1964 Declaration of Helsinki and its later amendments (World Medical Association, 2017) were followed. The studies were approved by the ethical committees of Turku University, Finland, and Nagoya University, Japan. Students completed the questionnaires anonymously during classes after their informed consent and were told that their privacy would be strictly protected. Guardian informed consent was obtained by informing them about the study and their possibility to refuse adolescent participation in the study. The questionnaires were collected in sealed envelopes by the teachers and sent to the researchers.

### Japanese cohort

The Japanese arm of the study was conducted in 2011, on Shikoku Island in the Tokushima Prefecture of Western Japan, which has 754,000 inhabitants and 96 schools. The study population included all 1,865 8th grade students, aged 13–14 years old, who were selected from the 17 junior high schools in the region and the participation was on a voluntary basis. The Tokushima Prefectural Board of Education selected these schools to be as representative as possible of the school population (geographical area, urbanicity, size of the school). All the students agreed to participate and a total of 1,840 questionnaires were included in the statistical analysis: 894 from male students and 946 from female students. The 25 questionnaires that were incomplete, or inappropriately filled in, were excluded from the analyses. The mean age of the respondents was 13.9 years, with a standard deviation (SD) of 0.2 years.

### Finnish cohort

The Finish study was conducted in 2014 in Rovaniemi, an area of Northern Finland with 61,000 inhabitants, and in Salo, an area of Southwestern Finland with 53,000 inhabitants. The study population included all 1,139 8th grade students aged 13–15 years old who attended the 13 junior high schools in the two regions on a certain school day. All junior high schools in Rovaniemi and Salo attended the study. The study sample is part of an on‐going time‐trend study examining changes in adolescents’ problem behavior (Sourander, Koskelainen, Niemelä, Rihko, Ristkari & Lindroos, 2012; Mishina, Tiiri, Lempinen, Sillanmäki, Kronström & Sourander, 2018). As previously reported, the study population from these two areas included both urban and suburban areas and the sex distribution, family composition and ethnic background were almost identical when we compared statistics for the same age group in the whole country (Sourander *et al*., 2012; Mishina *et al*., 2018). A total of 1,135 questionnaires were included in the statistical analysis, from 550 male students and 585 female students. The four questionnaires that were incomplete, or had been inappropriately filled in, were excluded from the analyses. The mean age of the students was 14.4 years (SD 0.5).

### Measures

A short nine‐item “Body Image and Eating Distress” scale developed by Koskelainen, Sourander & Helenius (2001) was used, which was designed to evaluate attitudes and behaviors towards dieting and body shape (Table 1). The items were “I would like to be thinner”; “I exercise a lot to avoid gaining weight”; “I have been on a diet”; “I am afraid of getting fat”; “I have lost weight”; “I am not happy with my body”; “It terrifies me if I gain even a little weight”; “I am not always able to control my eating”; “I devour large amounts of food at one time”. The Japanese version of the questionnaire was carefully translated from and back‐translated from the original one to ensure its accuracy. The items were scored zero if the statements were not true, one if they were somewhat true and two if they were certainly true. The sum of the items was used to generate an eating disorder total score ranging from zero to 18. The sum score has been shown to strongly correlate with the Strengths and Difficulties Questionnaire total score, emotional and behavioral subscores, alcohol use, body mass and help‐seeking in population‐based study including 1,458 adolescents (Koskelainen *et al*., 2001). The internal consistency between the items was good, with Cronbach's alpha of 0.83 in both the Japanese and Finnish samples. We did not include additional two items in the Japanese version, namely vomiting and the use of pharmaceutical drugs to control weight, because those questions were considered culturally inappropriate in Japan for a general school survey. However, these two items were used in Finnish questionnaire and their distributions for Finns are presented.

**Table 1:** Summary of multinomial logistic regression models. Comparison between Japanese and Finnish adolescents, with body image and eating behavior items as response variables

Response variable in multinomial logistic regression model	Gender													
	Females							Males						
	Country							Country						
	FIN (Ref.)		JPN					FIN (Ref.)		JPN				
	N	%	N	%	OR	95% CI	p‐value	N	%	N	%	OR	95% CI	p‐value
Item 1: I would like to be thinner^a^														
1: Not true	165	28.3	125	13.3	Ref.			343	62.5	601	68.6	Ref.		
2: Somewhat true	236	40.5	314	33.5	1.8	1.3 –2.4	**< 0.001**	159	29.0	211	24.1	0.8	0.6 – 1.0	**0.047**
3: Certainly true	182	31.2	499	53.2	3.6	2.7 – 4.8	**< 0.001**	47	8.6	64	7.3	0.8	0.5 – 1.2	0.217
Item 2: I exercise a lot to avoid gaining weight^a^														
1: Not true	188	32.30	423	45.0	Ref.			233	42.8	559	63.8	Ref.		
2: Somewhat true	319	54.81	400	42.6	0.6	0.4 – 0.7	**< 0.001**	238	43.7	245	28.0	0.4	0.3 – 0.5	**< 0.001**
3: Certainly true	75	12.89	117	12.5	0.7	0.5 – 1.0	**0.033**	74	13.6	72	8.2	0.4	0.3 – 0.6	**< 0.001**
Item 3: I have been on a diet^a^														
1: Not true	374	64.3	449	47.8	Ref.			497	90.7	783	89.5	Ref.		
2: Somewhat true	130	22.3	293	31.2	1.9	1.5 – 2.4	**< 0.001**	45	8.2	68	7.8	1.0	0.6 – 1.4	0.835
3: Certainly true	78	13.4	197	21.0	2.1	1.6 – 2.8	**< 0.001**	6	1.1	24	2.7	2.5	1.0 – 6.3	**0.043**
Item 4: I am afraid of getting fat^a^														
1: Not true	188	32.3	307	32.7	Ref.			418	32.7	619	70.6	Ref.		
2: Somewhat true	197	33.8	336	35.8	1.0	0.8 – 1.4	0.736	110	35.8	175	20.0	1.1	0.8 – 1.4	0.601
3: Certainly true	198	34.0	295	31.5	0.9	0.7 – 1.2	0.482	21	31.5	83	9.5	2.7	1.6 – 4.4	**< 0.001**
Item 5: I have lost weight														
1: Not true	452	77.5	816	87.0	Ref.			503	91.8	825	94.1	Ref.		
2: Somewhat true	86	14.8	62	6.6	0.4	0.3 – 0.6	**< 0.001**	35	6.4	40	4.6	0.7	0.4 – 1.1	0.129
3: Certainly true	45	7.7	60	6.4	0.7	0.5 – 1.1	0.141	10	1.8	12	1.4	0.7	0.3 – 1.7	0.469
Item 6: I am not happy with my body														
1: Not true	133	22.8	139	14.8	Ref.			325	59.2	459	52.3	Ref.		
2: Somewhat true	279	47.8	360	38.3	1.2	0.9 – 1.6	0.147	173	31.5	264	30.1	1.1	0.9 – 1.4	0.525
3: Certainly true	172	29.5	440	46.9	2.5	1.8 – 3.3	**< 0.001**	51	9.3	154	17.6	2.1	1.5 – 3.0	**< 0.001**
Item 7: It terrifies me if I gain even a little weight^a^														
1: Not true	320	54.9	579	61.7	Ref.			505	92.0	769	87.8	Ref.		
2: Somewhat true	168	28.8	251	26.8	0.8	0.7 – 1.0	0.115	33	6.0	69	7.9	1.4	0.9 – 2.1	0.148
3: Certainly true	95	16.3	108	11.5	0.6	0.5 – 0.9	**0.003**	11	2.0	38	4.3	2.3	1.1 – 4.5	**0.018**
Item 8: I am not always able to control my eating														
1: Not true	311	53.4	467	49.8	Ref.			403	73.4	596	68.0	Ref.		
2: Somewhat true	205	35.2	294	31.3	1.0	0.8 – 1.2	0.694	123	22.4	208	23.7	1.1	0.9 – 1.5	0.305
3: Certainly true	66	11.3	177	18.9	1.8	1.3 – 2.5	**< 0.001**	23	4.2	72	8.2	2.1	1.3 – 3.4	**0.003**
Item 9: I devour large amounts of food at one time^a^														
1: Not true	414	70.9	454	48.3	Ref.			353	64.4	554	63.2	Ref.		
2: Somewhat true	137	23.5	284	30.2	1.9	1.5 – 2.4	**< 0.001**	165	30.1	214	24.4	0.8	0.6 – 1.1	0.124
3: Certainly true	33	5.7	202	21.5	5.6	3.8 – 8.3	**< 0.001**	30	5.5	108	12.3	2.3	1.5 – 3.5	**< 0.001**
Item 10: I have wilfully vomited after having eaten^b^														
1: Not true	531	90.8	–	–	–	–	–	544	99.3	–	–	–	–	–
2: Somewhat true	29	5.0	–	–	–	–	–	3	0.6	–	–	–	–	–
3: Certainly true	25	4.3	–	–	–	–	–	1	0.2	–	–	–	–	–
Item 11: I have used pharmaceuticals to control my weight (appetite restricting drugs, laxatives or diuretics) b														
1: Not true	572	98.1	–	–	–	–	–	543	99.3	–	–	–	–	–
2: Somewhat true	9	1.5	–	–	–	–	–	4	0.7	–	–	–	–	–
3: Certainly true	2	0.3	–	–	–	–	–	0	0.0	–	–	–	–	–

Notes

Ref. = reference category. ^a^Country X Gender interaction was significant (*p* < 0.1) in a two–way model. ^b^Only collected in the Finnish study.

*P* values indicating statistically significance (*p* < 0.05) are shown in a bold type face.

The emotional, conduct, hyperactivity, peer and prosocial problems of the adolescents were evaluated with the adolescent self‐report version of the Strengths and Difficulties Questionnaire (SDQ). The SDQ includes 25 items, which deal with positive and negative behavioral traits, and these are scored with zero for not true, one for somewhat true and two for certainly true. The 25 items are divided into five scales – emotional symptoms, conduct problems, hyperactivity, peer problems and prosocial scales – that each contain five items. Both the Japanese and Finnish versions of the SDQ have been found to function well and their psychometric properties are adequate (Koskelainen, Sourander & Kaljonen, 2000; Noda, Ito, Harada*,* Nakajima, Takayanagi & Someki, 2013). In the Japanese survey, the teachers requested that the pupils were not asked about their height and weight.

### Statistical methods

Multivariate logistic regression analyses were used to compare genders and adolescents from Japan and Finland in the individual eating distress symptom items. Odds ratios (OR) and 95% confidence intervals (95% CI) were calculated. Gender and study group associations of eating distress sum scale were studied using 2‐way general linear models (GLM). Means, standard deviations and F‐test statistics are presented. More complicated comparison of gender, study country, separate SDQ subscales and their interactions in eating distress sum scale were studied with GLM as well. F‐test p‐values are shown. The 80th percentile cut‐off points of the scoring distribution in the present sample were used in the SDQ problem subscales to indicate borderline or clinical levels of symptoms (Goodman, 1997; Koskelainen *et al*., 2000). In case of each SDQ subscale, the optimal model was determined to start with a full three‐way model and reducing it as much as possible without statistically significant loss of determination, which was assessed with likelihood ratio tests. Three‐way interactions were studied further when they were significant. Pairwise least square means (LS means) and their differences for meaningful group comparisons were calculated and the Bonferroni correction coefficient m = 8 was used to handle Type I errors. All analyses were conducted using SAS version 9.4 software (SAS Institute Inc., Cary, NC).

## Results

Table 1 shows the results for body image and eating behavior items comparing separately for Japanese and Finnish males and females. The females from the two countries reported much more dissatisfaction and concerns with their bodies than the males. For example, only 14.8% of Japanese females and 22.8% of Finnish females were satisfied with their current body shape, compared to 52.3% of Japanese males and 59.2% of Finnish males. Japanese adolescents of both sexes had significantly higher levels of dissatisfaction with their body, dieting problems and difficulty controlling their appetite than Finnish males and females. A higher percentage of Japanese than Finnish females wanted to be thinner. Japanese males were more concerned about gaining weight and afraid of becoming fat than Finnish males. Finnish adolescents of both sexes reported higher levels of exercise to control weight than their Japanese peers. Finnish females were more terrified of weight gain than Japanese females. It was notable that 9.2% of Finnish females and 0.8% of Finnish males vomited to control their weight, a question that was not included in the Japanese survey (Table 1).

**Figure 1:**
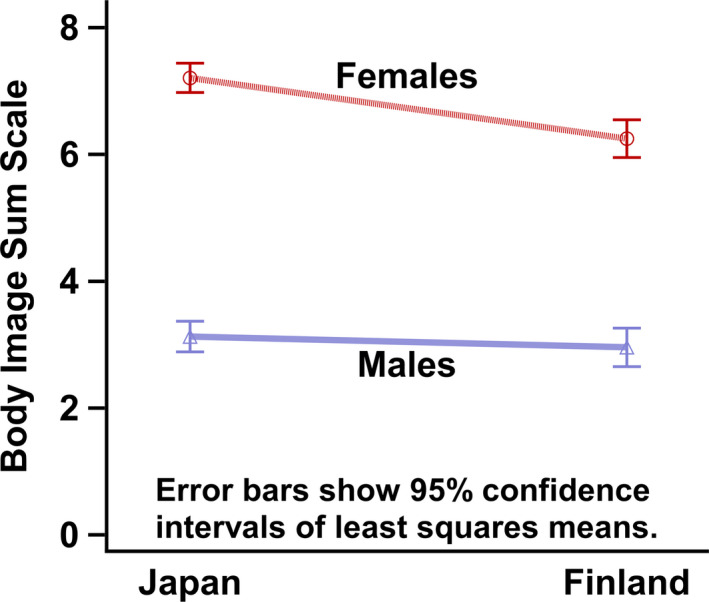
Least squares means and their 95% confidence intervals of body image and eating problems scale. Interaction plot of gender and country. [Colour figure can be viewed at wileyonlinelibrary.com]

Table 2 shows the differences in the LS means for total scores in body image and eating when the Japanese and Finnish students were compared. This showed that females from both countries had higher scores than males and Japanese females had higher mean scores than Finnish females. An interaction between country and gender was statistically significant (Fig. 1).

**Table 2:** Descriptive statistics and GLM analysis summary of body image and eating problem total sum scale for the Japanese and Finnish adolescents school study

GLM Summary				Least squares means							
Effect	F	Df	p		Finland		Japan			LS means difference	p
					LS mean	SE	LS mean	SE			
Gender	712.9	1	< 0.001	Females	6.25	0.152	7.21	0.119	Females: Japan–Finland	0.9581	< 0.001
Country	16.7	1	< 0.001	Males	2.96	0.155	3.13	0.123	Males: Japan–Finland	0.1703	0.390
Gender × Country interaction	8.1	1	0.004						Finland: Females–Males	3.2919	< 0.001
Error		2909							Japan: Females–Males	4.0797	< 0.001

Notes

LS mean = least squares mean; Df = degrees of freedom of the F test; SE = standard error.

**Figure 2:**
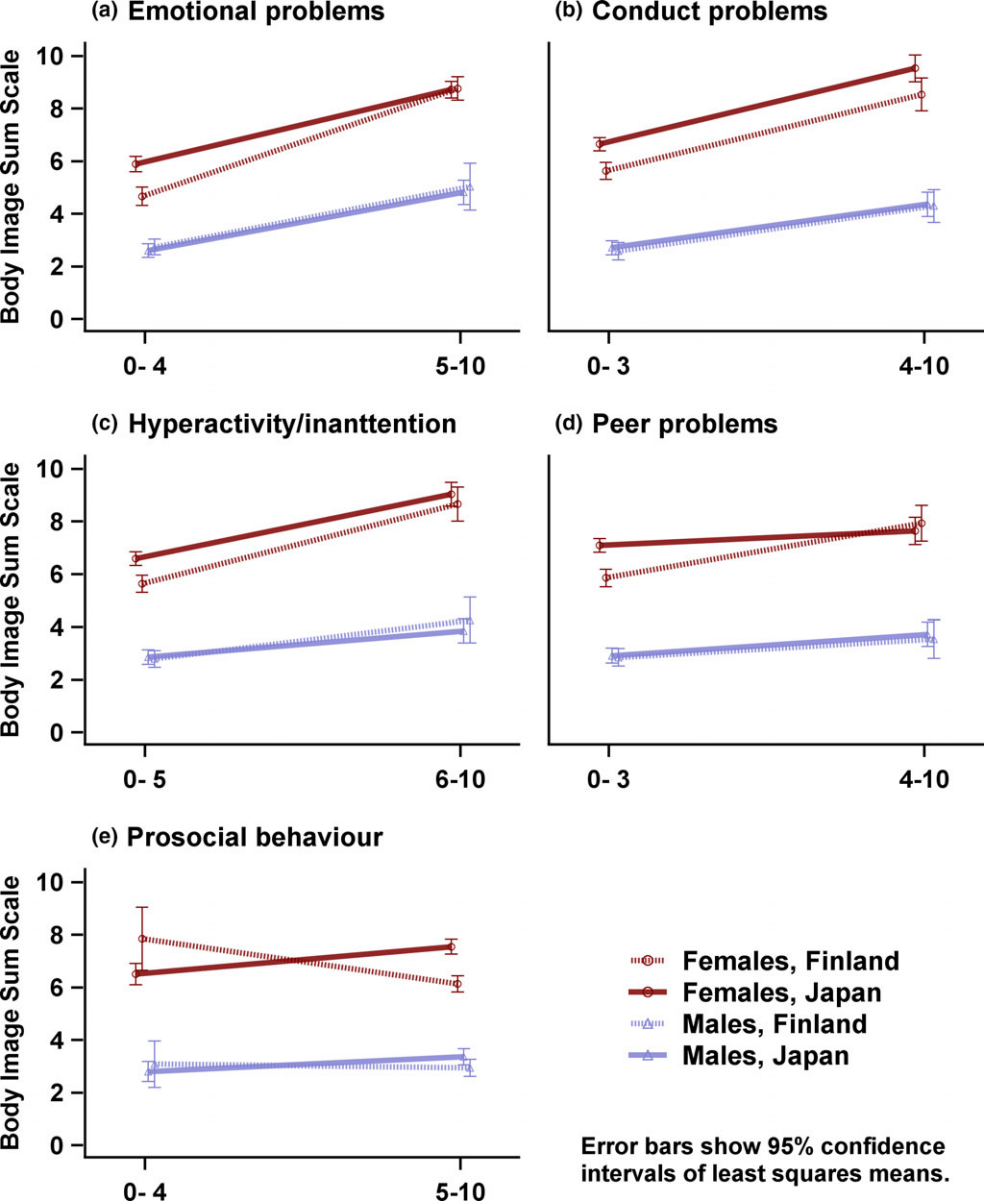
Least squares means and their 95% confidence intervals of body image and eating problems scale. Interaction plot matrix of gender, country and categorized SDQ subscales. [Colour figure can be viewed at wileyonlinelibrary.com]

Figure 2 shows LS means and their 95% confidence intervals when the total scores for body image and eating distress were compared between those who scored above and below the 80th percentile cut‐off point for each scale. As seen in Table 3, models that covered country, gender and SDQ scales with interactions, showed that the three‐way interaction effect of body image, gender and country was significant with peer problems (*p* = 0.017) and prosocial behavior (*p* = 0.012).

**Table 3:** Summary of GLM interaction models, with the body image and eating problem scale as the response and the SDQ subscales, gender and country as the predictors, for the Japanese and Finnish adolescents school study

GLM main and interaction effects degrees of freedom values, F‐values and *p*‐values.									
			Main effects			Two–way interactions			Three–way interaction
			SDQ subscale	Gender	Country	SDQ subscale × gender	SDQ subscale × country	Gender × country	SDQ subscale × gender × country
Model #: dichotomized SDQ subscale name	Num DF	Den DF	F p	F p	F p	F p	F p	F p	F p
1: Emotional problems	1	2893	300.23	379.76	1.63	13.44	4.20	5.32	3.23
			**< 0.001**	**< 0.001**	0.20	**< 0.001**	**0.041**	**0.021**	0.073
2: Conduct problems	1	2897	213.05	658.38	17.84	15.02	<Not included>	11.31	<Not included>
			**< 0.001**	**< 0.001**	**< 0.001**	**< 0.001**		**< 0.001**	
3: Hyperactivity	1	2894	138.58	616.19	9.28	23.41	<Notincluded>	9.33	<Not included>
			< 0.001	< 0.001	0.002	**< 0.001**		**0.002**	
4: Peer problems	1	2893	35.54	499.15	3.00	2.67	4.19	1.01	5.68
			**< 0.001**	**< 0.001**	0.084	0.10	**0.041**	0.32	**0.017**
5: Prosocial behavior	1	2893	0.08	333.85	0.05	1.59	15.88	0.01	5.59
			0.77	**< 0.001**	0.81	0.21	**< 0.001**	0.93	**0.012**

Notes

Num DF: numerator degrees of freedom of the F‐test; Den DF = denominator degrees of freedom of the F‐test. Models are selected using the likelihood ratio tests and the most reduced model without significant loss of determination is shown. P values indicating statistically significance (*p* < 0.05) are shown in a bold type face.

First, in the additional analyses, seen in Table 4, which included peer problems, the LS mean estimate of eating distress was higher among Japanese than Finnish females with low levels of peer problems, with a difference of 1.24 (*p* < 0.001). Finnish females with high levels of peer problems had more eating distress than Finnish females with low levels of peer problems (2.08, *p* < 0.001). However, similar differences were not found among Japanese females (0.55, *p* = 0.051). High levels of peer problems among Japanese males were associated with more eating distress, although the mean difference was rather small (0.81, *p* = 0.027). Similar statistically significant differences were not found among Finnish males (0.69, *p* = 0.73).

**Table 4:** GLM interactions. Least squares mean estimates and pairwise comparisons from interaction models. Response variable body image and eating problem scale. Japanese and Finnish adolescents school study

SDQ emotional symptoms (3‐way model)				
	LS means		LS means difference	P–value^a^
Females with low level of emotional symptoms: country difference	Finland	Japan		
	4.67	5.90	–1.23	**< 0.001**
Finnish females: difference in emotional symptoms	Score 0–4	Score 5–10		
	4.67	8.77	–4.10	**< 0.001**
Japanese females: difference in emotional symptoms	Score 0–4	Score 5–10		
	5.90	8.73	–2.83	**< 0.001**
Males with low level of emotional symptoms: country difference	Finland	Japan		
	2.74	2.61	0.13	~1.0
Finnish males: difference in emotional symptoms	Score 0–4	Score 5–10		
	2.74	5.04	–2.30	**< 0.001**
Japanese males: difference in emotional symptoms	Score 0–4	Score 5–10		
	2.61	4.83	–2.21	**< 0.001**
Females with high level of emotional symptoms: country difference	Finland	Japan		
	8.77	8.73	0.04	~1.0
Males with high level of emotional symptoms: country difference	Finland	Japan		
	5.04	4.83	0.21	~1.0
SDQ Conduct problems (reduced model)				
Females: difference in conduct problems	Score 0–3	Score 4–10		
	6.15	9.04	–2.89	**< 0.001**
Males: difference in conduct problems	Score 0–3	Score 4–10		
	2.65	4.33	–1.68	**< 0.001**
Females: country difference	Finland	Japan		
	7.09	8.10	–1.01	**< 0.001**
Males: country difference	Finland	Japan		
	3.44	3.55	–0.11	~1.0
SDQ Hyperactivity (reduced model)				
Females, difference in hyperactivity	Score 0–5	Score 6–10		
	6.14	8.78	–2.64	**< 0.001**
Males: difference in hyperactivity	Score 0–5	Score 6–10		
	2.85	3.95	–1.10	**< 0.001**
Females: country difference	Finland	Japan		
	7.04	7.87	–0.83	**< 0.001**
Males: country difference	Finland	Japan		
	3.40	3.40	0.00	~1.0
SDQ Peer relation problems (3‐way model)				
Females with low level of peer relation problems, country difference	Finland	Japan		
	5.86	7.10	–1.24	**< 0.001**
Finnish females: difference peer relation problems	Score 0–3	Score 4–10		
	5.86	7.94	–2.08	**< 0.001**
Japanese females: difference in peer relation problems	Score 0–3	Score 4–10		
	7.10	7.65	–0.55	0.51
Males with low level of peer relation problems: country difference	Finland	Japan		
	2.86	2.92	–0.07	~1.0
Finnish males: difference in peer relation problems	Score 0–3	Score 4–10		
	2.86	3.55	–0.69	0.73
Japanese males: difference in peer relation problems	Score 0–3	Score 4–10		
	2.92	3.73	–0.81	**0.027**
Females with high level of peer relation problems: country difference	Finland	Japan		
	7.94	7.65	0.29	~1.0
Males with high level of peer relation problems: country difference	Finland	Japan		
	3.55	3.73	–0.18	~1.0
SDQ Prosocial behavior (3‐way model)				
Females with low prosocial behavior level: country difference	Finland	Japan		
	7.86	6.51	1.34	0.29
Finnish females: difference in prosocial behavior level	Score 0–4	Score 5–10		
	7.86	6.15	1.71	0.052
Japanese females: difference in prosocial behavior level	Score 0–4	Score 5–10		
	6.51	7.55	–1.04	**< 0.001**
Males with low prosocial behavior level: country difference	Finland	Japan		
	3.09	2.81	0.28	~1.0
Finnish males: difference in prosocial behavior level	Score 0–4	Score 5–10		
	3.09	2.95	0.14	~1.0
Japanese males: difference in prosocial behavior level	Score 0–4	Score 5–10		
	2.81	3.37	–0.56	0.20
Females with high prosocial behavior level: country difference	Finland	Japan		
	6.15	7.55	–1.41	**< 0.001**
Males with high prosocial behavior level: country difference	Finland	Japan		
	2.95	3.37	–0.42	0.54

Notes

a P–value for pairwise different using Bonferroni multiple comparison adjustment with correction coefficient 8 (3‐way models) or 4 (reduced models). LS mean: Least squares mean. P values indicating statistically significance (*p* < 0.05) are shown in a bold type face.

Second, the additional analysis also studied similar interactions with prosocial behavior. Japanese females with good prosocial behavior scored higher on the eating problem scale – indicating more problems – than Japanese females with lower prosocial scores, which indicated poorer prosocial skills (1.04, *p* < 0.001). The opposite trend was observed with Finnish females and this result was close to significance (1.71, *p* = 0.052), indicating that eating distress were associated with poorer prosocial skills. Japanese females with good prosocial behavior had more eating distress than Finnish females with good prosocial behavior (1.41, *p* < 0.001). Figures 2D and 2E illustrate these differences in the peer and prosocial scales.

## Discussion

The findings show both similarities and differences in body image and eating distress between Japanese and Finnish adolescents. First, females in both Finland and Japan reported much higher distress than male adolescents in the two countries. Second, Japanese females reported more distress than Finnish females when it came to dissatisfaction with their body image and difficulty in controlling their appetite. Third, the general picture that emerged was that body image and eating distress levels showed greater differences between the genders in Japan than in Finland. However, gender had a higher impact on eating distress than nationality. Fourth, psychological problems were associated with body image and eating distress.

The finding that female adolescents experienced more distress than males in both Japan and Finland was in accordance with previous studies that found that body image and eating distress were more common among females (Hannan, Perry & Irving 2002; Presnell, Bearman & Stice, 2004; Neumark‐Sztainer, Story, Stice & Whitenton, 2002). Having a slender ideal body image and experiencing eating distress has mainly been confirmed in Caucasian females living in Western countries.

An important finding was that Japanese females experienced higher distress levels with regard to their body image and eating behavior than Finnish female adolescents. In a comparative study on body image and weight control among college students in 22 countries, Japanese college students showed the highest incidence of overestimating weight and weight control attempts (Wardle, Haase & Steptoe, 2006). In the last decade, the average weight of young Japanese adolescents has gradually been getting lower and the rates for adolescents who are thin and too thin have been increasing, reversing the increasing trends for overweight and obesity up to the mid‐2000s (Cabinet Office, Government of Japan, 2013). It is common for adolescents to overestimate their body size and want to be slender, irrespective of their actual size. The processes of modernisation and westernisation, including exposure to media and ultra‐thin models and celebrities, may raise body image problems (Rieger *et al*., 2001). Chisuwa and O'Dea (2010) stated that personal factors, such as low self‐esteem and social anxiety, appeared to be culturally mediated influential factors that diminished the body image of Japanese youth. Collectivist cultures like Japan, which value the needs of the community over the individual, have been shown to place a strong emphasis on conformity to social norms (Nogami, 1997). In such cultures self‐assertion is considered as immature or selfish, and praising and promoting oneself is regarded as bad manners (Kayano *et al*., 2008). Changing gender roles are also important in countries like Japan, in addition to the country's socio‐cultural aspects. Japan performed very badly in the Global Gender Gap Index 2015, was ranked 101st out of 145 countries, the second least gender equal society of the industrialized countries. Women are still expected to take a step back from men and to have the traditional female role in Japanese society (Chisuwa & O'Dea, 2010). However, taking this socio‐cultural standardized gender role, Japanese females may have lower self‐esteem and lower ability to assert themselves and cope with social issues (Pike & Borovoy, 2004). This low self‐esteem and expected self‐deprecation, combined with strongly held views that in order to be beautiful Japanese women need to be slim, may lead to Japanese women experiencing issues with their body image and eating distress (Saito, 2004).

This study shows that Japanese and Finnish male adolescents had more similar prevalence rates than females when it came to body image and eating behavior. A number of studies have reported that females experience more body image issue and eating distress than males and only small differences in body image and in weight control activities have been reported across different cultural groups (Viner *et al*., 2006). However, most of the questionnaires that have been developed to address body image concerns have been designed for females, and, as a result, they may not be able to evaluate a male's desire for increased bulk or muscle (McCabe & Ricciardelli, 2004), which should be considered in future research. Studies have reported that body image distress among males has increased over the last few decades (Cohane & Pope, 2001; McCabe & Ricciardelli, 2004).

The finding in this study that body image and eating distress was associated with emotional and behavioral problems has also been shown in previous studies (Ferreiro *et al*., 2012; Koskelainen *et al*., 2001; Mäkinen *et al*., 2012; Paxton *et al*., 2006). Overall, our findings suggest that there were different associations between peer and prosocial behavior and body image and eating distress among Japanese and Finnish females. Interestingly, better prosocial behavior among Japanese females was associated with more eating distress compared to those who had poorer prosocial behavior. Japanese females with better prosocial behavior had also more eating distress than Finnish females with better prosocial behavior. Similarly, Japanese females with low level of peer problems had more eating distress than Finnish females with low level of peer problems. These findings indicate that associations between peer and prosocial behavior and body image and eating distress are partially mediated by culture. One possible interpretation is that there is more pressure on Japanese female to be thin than Finnish females. Pike and Borovoy (2004) suggest that for Japanese females losing weight may not associated with the desire to achieve an unrealistic beauty ideal, but it is rather associated with wanting to conform to contemporary social norms of appearance among their peer group – pursuing “sameness” that reflects the pressure of the collectivist culture. In the present study good social competence among Japanese female adolescents seemed to be paradoxically associated with more distress in body image and eating problems. However, the study design did not enable us to examine the direction of the causalities of these findings.

This study compared adolescents of the same age in two culturally diverse countries with very different attitudes towards the standing and roles of females. Most of the existing research about eating distress or pathology has come from the Western world and extrapolating these results, without any comparable data, is unlikely to present a true global picture of these problems. In order to address the ever‐increasing global burden of eating distress faced by adolescents, we need collaborative research from both Western and non‐Western countries. Similar studies are warranted, that compare adolescents from different parts of the world, from countries with different levels of human development and from societies with different cultural conceptions of female roles.

### Limitations

There were several limitations with this study that should be taken into consideration. First, although the samples were relatively large, they represented certain geographical areas in Japan and Finland. Second, there was a risk of misinterpretation or cultural differences in the way that respondents perceived questions about body image and eating distress. Third, the study was based on two cross‐sectional surveys using self‐reports and it was not possible to study the causality, for example between psychopathology and body image and eating distress. Furthermore, it is always possible that there are cultural differences when it comes to subjects reporting distress and behavioral problems. Of note, our aim was not to examine anorexia or bulimia in the population. The questionnaire focused on attitudes and behaviors towards dieting and body shape. Unfortunately, the “Body image and Eating Distress” questionnaire was not validated using clinical samples. However, in our cross‐cultural school survey, we wanted to use a brief questionnaire that would suit both Finnish and Japanese cultures. For this reason, we used this nine‐item questionnaire which was suitable for the needs of both countries and which was also well‐established in our previous clinical use. The questionnaire was originally developed as part of a European Cooperation in Science and Technology (COST) project (2011). Of note, all specific items were very similar to those included in e.g. Eating Disorders Inventory (Garner, 1991; Mustelin, Kärkkäinen, Kaprio & Keski‐Rahkonen, 2016), which is a much longer questionnaire but has been extensively validated. It is important to acknowledge that dieting and body image problems have a wider significance in adolescent well‐being globally beyond the clinical diagnoses. Our study had considerable strengths. The cohort size from the two countries was almost 3,000 students aged 13–15, with almost equal numbers of males and females. The surveys were completed in a controlled classroom setting, with guaranteed privacy and confidentiality, and this generated a very low percentage of unusable responses, of less than 1%. It also compared two very diverse societies: Finland, which came third in the Global Gender Gap Index 2015 and Japan, which was almost last in the list of industrialised countries. This provided a strong international context for the differences between females in the two country's cohorts.

## Conclusions

Distress related to body image and eating were very high among female adolescents in Finland and were even higher in Japan. High levels of distress with body image and eating were associated with emotional and behavioral problems. The country‐based differences between the males in the two cohorts were less significant.
